# Spatial Changes in the Atrial Fibrillation Wave-Dynamics After Using Antiarrhythmic Drugs: A Computational Modeling Study

**DOI:** 10.3389/fphys.2021.733543

**Published:** 2021-09-24

**Authors:** Inseok Hwang, Ze Jin, Je-Wook Park, Oh-Seok Kwon, Byounghyun Lim, Jisu Lee, Hee-Tae Yu, Tae-Hoon Kim, Boyoung Joung, Hui-Nam Pak

**Affiliations:** Yonsei University Health System, Seoul, South Korea

**Keywords:** atrial fibrillation, computational modeling, antiarrhythmic drug, dominant frequency, spatial changes

## Abstract

**Background:** We previously reported that a computational modeling-guided antiarrhythmic drug (AAD) test was feasible for evaluating multiple AADs in patients with atrial fibrillation (AF). We explored the anti-AF mechanisms of AADs and spatial change in the AF wave-dynamics by a realistic computational model.

**Methods:** We used realistic computational modeling of 25 AF patients (68% male, 59.8 ± 9.8 years old, 32.0% paroxysmal AF) reflecting the anatomy, histology, and electrophysiology of the left atrium (LA) to characterize the effects of five AADs (amiodarone, sotalol, dronedarone, flecainide, and propafenone). We evaluated the spatial change in the AF wave-dynamics by measuring the mean dominant frequency (DF) and its coefficient of variation [dominant frequency-coefficient of variation (DF-COV)] in 10 segments of the LA. The mean DF and DF-COV were compared according to the pulmonary vein (PV) vs. extra-PV, maximal slope of the restitution curves (Smax), and defragmentation of AF.

**Results:** The mean DF decreased after the administration of AADs in the dose dependent manner (*p* < 0.001). Under AADs, the DF was significantly lower (*p* < 0.001) and COV-DF higher (*p* = 0.003) in the PV than extra-PV region. The mean DF was significantly lower at a high Smax (≥1.4) than a lower Smax condition under AADs. During the episodes of AF defragmentation, the mean DF was lower (*p* < 0.001), but the COV-DF was higher (*p* < 0.001) than that in those without defragmentation.

**Conclusions:** The DF reduction with AADs is predominant in the PVs and during a high Smax condition and causes AF termination or defragmentation during a lower DF and spatially unstable (higher DF-COV) condition.

## Introduction

Atrial fibrillation (AF) is a common arrhythmia, with a prevalence of more than 1.6% of the total population, and the prevalence continues to increase in the aging society (Kim et al., 2018). Antiarrhythmic drugs (AADs) are the most commonly used first-line treatment for AF rhythm control. However, inadvertent use of AADs can increase the mortality (Cardiac-Arrhythmia-Suppression-Trial-(CAST)-Investigators, 1989; The-AFFIRM-Investigators, 2004) and has the risk of various side effects (Chandhok and Schwartzman, 2007). After the establishment of the guidelines of AF management on the use of AADs, the safety of AADs has been improving, and early rhythm control using AADs ensures a better prognosis in AF patients (Kirchhof et al., 2020; Hindricks et al., 2021). Nevertheless, as AADs are ion channel blockers, their efficacy highly varies from person to person due to the interaction of multiple ion channels and the genetic influence (Darbar and Roden, 2013) and remains unsatisfactory (Roy et al., 2000). Many experimental studies have conducted to investigate the effects of AADs, however, most of the studies were results of animal studies (Varela et al., 2016). Previous study indicated that APD heterogeneity promoted substrate for arrhythmogenic re-entrant waves during AF initiation and maintenance. Amiodarone has shown anti-AF effect by increasing atrial APD and reducing APD heterogeneity. Increasing atrial APD and reduced APD heterogeneity were effective in controlling arrhythmogenic reentry (Varela et al., 2016). If the rhythm control effect of AADs can be predicted through simulation modeling, an efficient selection of AADs might be possible and can reduce the adverse effects or trial and error. We recently reported that the virtual AAD test can be performed through computational modeling reflecting the personalized atrial anatomy, histology, and electrophysiology of AF patients (Hwang et al., 2021). Computational modeling can evaluate the efficacy of multiple AADs under the same condition and can quantify the mechanistic effects of AADs using very high-spatiotemporal resolution maps (Loewe et al., 2014; Li et al., 2016; Lim et al., 2020b; Bai et al., 2021; Hwang et al., 2021). This virtual AAD test does not have any ethical problems because it allows testing of multiple drugs with variable doses without the risk of adverse events (Hwang et al., 2021). This study analyzed the mechanism of the AAD effects on the AF wave-dynamics using an AF computational modeling that reflected the anatomical, histological, and electrophysiological characteristics of 25 patients with AF. The purpose of this study was to quantify the dominant frequency (DF) and its spatial heterogeneity after using AADs (Jarman et al., 2012; Kogawa et al., 2015; Li et al., 2016) and to compare the regional differences between the pulmonary veins (PVs) and extra-PV regions and differences according to the AF wave-break conditions (Li et al., 2016). In addition, we compared the characteristics of the wave-dynamics in episodes of AF termination or defragmentation under AAD use.

## Materials and Methods

### Ethical Approval

The study protocol followed the Declaration of Helsinki and was approved by the Institutional Review Board of the Severance Cardiovascular Hospital, Yonsei University Health System. All participants were included in Yonsei AF Ablation Cohort Database (ClinicalTrials.gov Identifier: NCT02138695) and provided written consent to participate in the study.

### Activation Time Matching

**FIGURE 1:**
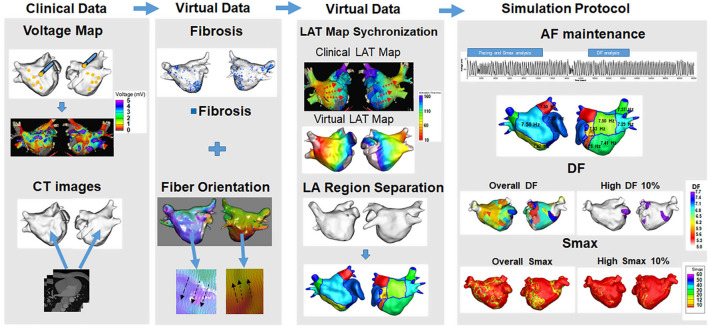
Computational modeling of the left atrium with atrial fibrillation (AF). Realistic left atrium (LA) modeling was conducted using an interpolation of the voltage map and merging with the CT images. Fibrosis and the fiber orientation were implemented. The LAT map synchronization and AF simulation protocol were conducted for the analyses.

First, electroanatomical modeling using patient voltage data was conducted. Over 500 bipolar voltage data points that included sequential recordings of electrograms at a 500 ms cycle length were obtained from the surface of the individual atria during AF catheter ablation (Figure 1). The individual CT images were merged with the voltage data to produce the personalized electroanatomical environment of each patient. The inverse distance weighting (IDW) method (Ugarte et al., 2015) was used to interpolate the clinical voltage signal for a simulation. The interpolation was based on the IDW method (Ugarte et al., 2015) and was within a 10-mM radius from the region of interest. Interpolation of the clinical voltage data produced a virtual voltage map with an amplitude. The detailed equation for the IDW was as follows:


Wi⁢j=di⁢j-a∑knjdkj,Rj=∑i=1njwi⁢j⁢Ri⁢j

where W demonstrated the weighted average of neighboring values; i and j represented the unknown and known values of the respective points; d_*ij*_^–*a*^ was the distance between unknown and known points; R_*j*_ represented the interpolation value at unknown point j; and R_*ij*_ indicated the known point of the value. The 3D left atrium (LA) model was created using the interpolated voltage map and CT images through the Ensite Navx system (Abbott Inc., Lake Bluff, IL, United States). Accurate matching of the voltage and CT images data on the 3D LA model was conducted using rotation and translation. We interpolated a clinical voltage map to produce a virtual voltage map on 3D model. The registration of the electro-anatomical maps onto the CT models involved the four following steps: geometry, trimming, field scaling, and alignment. The registration error could occur during such steps. Each step was conducted manually by an operator therefore possible human error potentially existed (Lim et al., 2020a). The fiber orientation involved two states: tracking and visualization. Tracking was a parallel task making it effective for graphic processing unit (GPU)-based fiber tracking. The conductivity varied due to the direction of the vector. A vector pointing perpendicular to the direction of conductivity indicated slower conductivity compared with a vector pointing the same direction as the conductivity. The fiber orientation was produced by simulating a clinical local activation map as well as the atlas-based mesh of atrial geometry. The fiber orientation was produced by simulating a clinical local activation map as well as the atlas-based mesh of atrial geometry (Pashakhanloo et al., 2016; Lim et al., 2020a). We estimated personalized fiber orientations using an atlas-based method (Niederer et al., 2019; Roney et al., 2021) to reflect anisotropic conduction from isotropic triangular mesh with 300 μM edges. Then, we adjusted the fiber orientation based on the clinical LAT map. The conductivity of our model (Zahid et al., 2016) was applied 0.1264 S/m (non-fibrotic longitudinal cell), 0.0546 S/m (fibrotic longitudinal cell), 0.0252 S/m (non-fibrotic transverse cell), and 0.0068 S/m (fibrotic transverse cell). Fibrosis areas were estimated based on the clinical bipolar map. Fibrosis was determined using a non-linear relationship of the bipolar voltage and the probability of fibrosis. The equation for the probability of fibrosis was described as follows (Hwang et al., 2019):


$$P_{f\,i\,b\,r\,o\,s\,i\,s}=\left\{ \begin{array}{ccc} 1, & X<0 & \\ -40.0\,X^{3}+155\,X^{2}-206\,X+99.8\; & 0\leq X\leq 1.74 & \\ 0, & 1.74<X & \end{array}\right.$$

where X is the bipolar voltage at each node, and it was ranged from 0 to 1.74 mV. If X is >1.74 mV, then *P*_*fibrosis*_ would be zero. The probability of fibrosis was determined using clinical bipolar voltage data.

Fiber tracking was performed to determine the direction of the conduction. Fibrosis was represented using the relationship between the probability of fibrosis and bipolar voltage values (Zahid et al., 2016). The diffusion coefficient was calibrated by synchronization of the clinical and virtual conduction velocity. Before a preliminary simulation, conduction velocity was calculated by using the distance from the pacing location to the LA appendage and divided it by the travel time to get the conduction velocity. We then matched conduction velocity from the simulation to clinical conduction velocity by modulating the diffusion coefficient (Lim et al., 2020a). A color scale indicating the conduction time was compared between the clinical and virtual activation time maps for matching to produce an accurate conduction environment for each patient.

### Virtual Antiarrhythmic Drug Intervention

The human atrial myocyte model (Courtemanche et al., 1998) was used for normal sinus rhythm, and an AF state was created by modifying that model (Lee et al., 2016). For the baseline AF state, the I_*Na*_, I_*to*_, I_*CaL*_, I_*Kur*_, and I_*Caup*_ were decreased by 10, 70, 70, 50, and 20%, and the I_*K*__1_ was increased by 110% as compared to that of the Courtemanche model (Lee et al., 2016). Five types of AADs were used for the study. Class III included amiodarone, sotalol, and dronedarone, and class IC indicated flecainide, and propafenone. High dose included amiodarone 10 μM, sotalol 10 mM, dronedarone 10 μM, flecainide 15 μM, and propafenone 10 μM. Low dose included amiodarone 5 μM, sotalol 60 μM, dronedarone 3 μM, flecainide 5 μM, and propafenone 5 μM. All the ionic changes for each drug were derived from previously reported references. Our AAD references used IC50 values. We used such references and make percent changes relative to the Courtemanche-Ramirez-Nattel model (Courtemanche et al., 1998). The reduction of channel conductance was calculated to reflect the ion channel blocking effect at the considered concentration. For the implementation of ion currents for each dose, we conducted the literature search and implemented such information to construct the ion currents for each dose as previously reported in our study (Hwang et al., 2021). As the Courtemanche-Ramirez-Nattel model (Sossalla et al., 2010; Grandi et al., 2011) being the baseline, the effects of each dose were implemented by applying the blockage of specific ion channels. Supplementary Tables 1, 2 showed detailed descriptions of the ion current changes from baseline in response to the different AADs references including the class IC and class III drugs as well as each dose.

### Atrial Fibrillation Induction, Dominant Frequency, and Smax Analyses

Our GPU-based customized software (CUVIA ver. 2.5, Model: SH01; Laonmed Inc., Seoul, South Korea) was used virtually to induce and apply appropriate ion currents for AADs. The DF and Smax were analyzed using this same GPU-based software. AF was initiated in a pacing location using AF pacing from 200 to 120 ms with eight beats per cycle using ion currents for specific AADs. Virtual pacing location was matched with clinical activation time map for a realistic LA modeling. Before AF induction simulation, clinical and virtual pacing sites were matched to reflect the personalized LA model. Successful AF induction was determined during AF pacing by observing electrogram in the 3D LA map (Supplementary Figure 1). Defragmentation of AF includes termination of AF and conversion of AF to atrial tachycardia. Defragmentation was determined by visually assessing the electrogram and 3D activation map of each case. If there were <2 spiral waves, we determined it as a defragmented state. Once AF was induced successfully, maintenance of AF was observed up to 32 s. During the maintenance period, the DF was calculated from 17 to 23 s. APD_90_ was a normal sinus rhythm measured at a pacing cycle length of 600 ms. We calculated using non-linear fitting of APD_90_ and diastolic interval (Shattock et al., 2017) from over 400,000 nodes during single-site pacing. Smax values were defined at every node in LA regions per patient. For the regional analyses of the Smax and DF, the LA was divided into 10 regions. 10 regions of LA were decided based on the previous clinical study (Park et al., 2009). We used 3D spiral CT images of LA to divide LA portions according to the embryological origin. The portions include the venous LA (posterior LA including the antrum and posterior wall), anterior LA (excluding LA appendage), and LAA. We also divided PV antrum, posterior inferior wall, and septum along the posterior inferior line and septal line. The mean DF and mean Smax were calculated using the results of all 10 regions. A high DF and high Smax were defined as the respective top 10% of the values (Supplementary Figures 2, 3). For the stability of the DF and Smax after AADs, the coefficient of variation (COV) of the high DF and high Smax were calculated as the standard deviation divided by the mean:


$$C\,O\,V=\frac{\sigma }{\bar{x}}$$

σ represented the standard deviation, and $\bar{x}$ indicated the mean value.

### Statistical Analyses

The continuous variables were represented as the median and interquartile range. A comparison of the DF, Smax, and COV was conducted using a *t*-test and Mann-Whitney test depending on the distribution. A *p*-value < 0.05 was considered statistically significant. Any case in which the DF terminated before 17 s was excluded from the study. Statistical analyses were conducted using SPSS (IBM Corp., IBM SPSS Statistics for Windows, Version 21.0) and RStudio [RStudio Team (2020). RStudio: Integrated Development for R. RStudio, PBC, Boston, MA]^1^ software.

## Results

### Effects of Antiarrhythmic Drugs on the Atrial Fibrillation Wave-Dynamics

**FIGURE 2:**
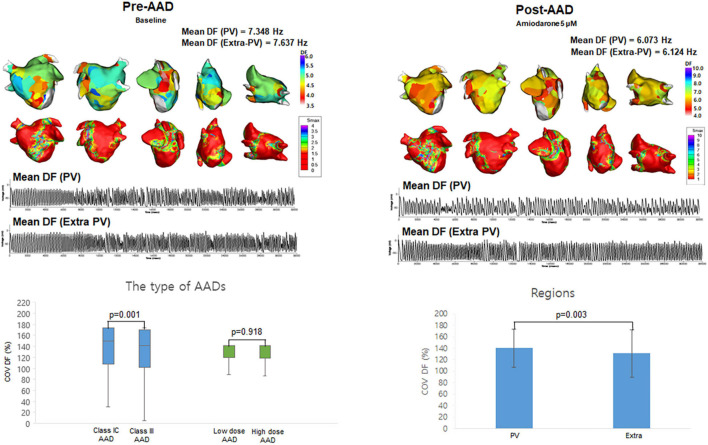
The effects of antiarrhythmic drugs (AADs) on extra-PV and pulmonary vein (PV) regions. The 3D dominant frequency (DF) map indicated that the mean DF was higher in the PV regions. Electrograms demonstrated regional voltage changes in PV and extra-PV areas. Coefficient of Variation-Dominant Frequency (COV-DF) was higher in class IC and PV region.

The patient group consisted of 25 AF patients (68.0% male, 59.8 ± 9.8 years old, 32.0% paroxysmal AF) who had undergone radiofrequency catheter ablation (Supplementary Table 3). Table 1 compared the effects of AADs on the electrophysiological parameters and wave-dynamic parameters. Overall (2 class IC and 3 class III AADs, low and high doses for each drug), the AADs prolonged the action potential duration for the 90% repolarization (APD_90_, *p* < 0.001) and mean AF cycle length (AFCL, *p* < 0.001) and reduced the conduction velocity (CV, *p* = 0.007), but did not change the Smax (*p* = 0.899). The DF (*p* < 0.001) and COV-DF (*p* = 0.001, Figure 2) reduction effects of the class III AAD were more significant than those of the class IC AADs (Table 1). AADs dose-dependently changed the APD_90_ (*p* < 0.001), AFCL (*p* < 0.001), and CV (*p* < 0.001), and the DF reduction was more pronounced at high doses than low doses (*p* < 0.001, Table 1).

**TABLE 1:** Effects of antiarrhythmic drugs (AADs) on the electrophysiological and fibrillatory wave-dynamics parameters.

	**Baseline (^†^*n* = 25)**	**Overall AADs (^†^*n* = 250)**	***P*-value**	**Class IC AADs (^†^*n* = 100)**	**Class III AADs (^†^*n* = 150)**	***P*-value**	**Low dose AADs (^†^*n* = 125)**	**High dose AADs (^†^*n* = 125)**	***P*-value**
APD_90_ (ms)	233.000 (231.000, 239.000)	273.000 (263.000, 295.000)	<0.001	269.000 (256.000, 290.000)	277.000 (265.000, 303.000)	0.002	267.000 (261.000, 273.000)	293.000 (271.000, 309.000)	<0.001
CV (m/s)	0.750 (0.617, 0.906)	0.612 (0.411, 0.741)	0.007	0.598 (0.474, 0.732)	0.618 (0.395, 0.745)	0.615	0.674 (0.484, 0.826)	0.526 (0.346, 0.685)	<0.001
Mean AFCL (ms)	135.616 (130.526, 150.303)	159.344 (145.632, 176.964)	<0.001	156.508 (140.000, 171.579)	162.312 (150.923, 183.952)	0.038	153.750 (140.000, 172.281)	164.167 (156.508, 192.404)	<0.001
Mean Smax	0.785 (0.656, 0.963)	0.802 (0.635, 1.009)	0.899	0.851 (0.639, 1.027)	0.744 (0.629, 0.998)	0.136	0.730 (0.628, 0.916)	0.851 (0.677, 1.106)	0.003
Mean DF (Hz)	7.025 (6.097, 7.379)	5.722 (1.286, 6.553)	<0.001	6.148 (5.315, 6.922)	5.170 (1.200, 6.145)	<0.001	6.121 (5.082, 6.874)	5.101 (1.200, 6.098)	<0.001
COV-DF (%)	NA	141.421 (105.265, 173.205)	NA	149.079 (108.095, 173.205)	141.421 (102.270, 170.349)	0.001	141.000 (120.000, 141.000)	141.000 (119.000, 141.000)	0.918

*APD_90_, Action potential duration 90%; CV, Conduction velocity; AFCL, AF cycle length; Smax, The Maximal slope of the restitution curves; DF, Dominant frequency; COV-DF, Coefficient of Variation-Dominant Frequency.*

*Patients who did not sustain proper normal sinus rhythm and an atrial fibrillation (AF) status were excluded from the analysis.*

*Median (IQ1, IQ3) was displayed in the Table.*

*^†^n = The number of patients × AAD × Dose.*

### Different Antiarrhythmic Drug Effects on the Pulmonary Vein and Extra-Pulmonary Vein Regions

Among the 10 segments of the LA, we compared the areas of the PV antrum and extra-PV regions (Table 2). The Smax and DF did not differ between the PV antrum and extra-PV LA regions during the baseline AF. After the administration of the AADs, the mean DF became lower (*p* < 0.001, Figure 2) and COV-DF higher (*p* = 0.003, Figure 2) at the PV antrum than in extra-PV LA regions, which suggested a lower and unstable DF on the PV antrum after AADs.

**TABLE 2:** Effects of antiarrhythmic drugs (AADs) on the pulmonary vein (PV) vs. Extra-PV tissue.

	**Baseline**			**AAD**		
	**PV (^†^*n* = 25)**	**Extra-PV (^†^*n* = 25)**	***P*-value**	**PV (^†^*n* = 750)**	**Extra-PV (^†^*n* = 500)**	***P*-value**
Mean Smax	1.258 (1.060, 1.619)	1.418 (1.006, 1.729)	0.541	1.264 (0.802, 1.659)	1.290 (0.892, 1.663)	0.541
ΔMean Smax	NA	NA	NA	−0.027 (−0.219, 0.170)	0.006 (−0.326, 0.250)	0.692
Mean DF (Hz)	7.567 (6.246, 8.186)	7.916 (7.383, 8.595)	0.086	6.464 (5.246, 7.170)	7.029 (6.209, 7.659)	<0.001
ΔMean DF	NA	NA	NA	−0.820 (−1.275, −0.236)	−0.848 (−1.368, −0.298)	0.238
COV-DF (%)	NA	NA	NA	141.421 (117.963, 173.205) ^‡^140.446 ± 33.227	141.421 (97.825, 172.515) ^‡^130.932 ± 41.633	0.003

*Smax, The Maximal slope of the restitution curves; ΔMean Smax, Changes of Smax; DF, Dominant Frequency; ΔMean DF, Changes of DF; COV-DF, Coefficient of Variation-Dominant Frequency.*

*Patients who did not sustain proper normal sinus rhythm and an atrial fibrillation (AF) status were excluded from the analysis.*

*Median (IQ1, IQ3) was displayed in the Table.*

*^†^n = The number of patients × AAD × Dose.*

*^‡^Mean ± SD.*

### Post-antiarrhythmic Drug Mean Dominant Frequency Depending on the Smax

**FIGURE 3:**
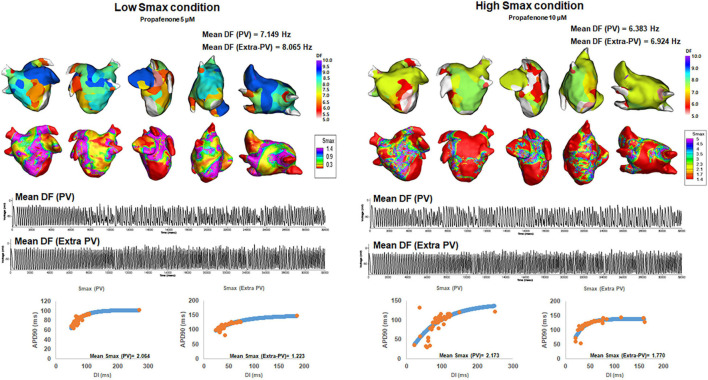
Changes in the dominant frequency (DF) during a high and low Smax. The 3D DF map indicated that the mean DF was inversely related to the mean Smax. Regional voltage changes in pulmonary vein (PV) and extra-PV areas were demonstrated in electrograms.

We compared the changes in the mean DF and COV-DF at a Smax value of 1.4, based on a previous clinical study for human atrial restitution (Table 3). In Table 3, we used the baseline Smax values threshold for baseline mean DF, and post-AAD Smax threshold for post-AAD mean DF. At a Smax ≥1.4, the post-AAD mean DF was significantly lower than that at a Smax <1.4 (*p* = 0.014, Figure 3). The pattern of a higher mean DF during a Smax <1.4 condition was consistent in the PV (*p* = 0.039, Figure 3) and extra-PV areas (*p* = 0.002, Figure 3). However, the COV-DFs did not differ depending on the Smax value. Additionally, we differentiated especially Table 3 into subgroups as indicated in Supplementary Table 4. DF was higher in dronedarone 3 μM and amiodarone 5 μM at low Smax.

**TABLE 3:** Atrial fibrillation (AF) wave-dynamics depending on the Smax values.

	**Overall**		***P*-value**	**PV**		***P*-value**	**Extra-PV**		***P*-value**
			
	**Smax < 1.4 (^†^*n* = 13)**	**Smax ≥ 1.4 (^†^*n* = 12)**		**Smax < 1.4 (^†^*n* = 13)**	**Smax ≥ 1.4 (^†^*n* = 12)**		**Smax < 1.4 (^†^*n* = 13)**	**Smax ≥ 1.4 (^†^*n* = 12)**	
Baseline mean DF (Hz)	7.958 (7.138, 8.485)	7.708 (7.194, 8.013)	0.650	7.797 (6.246, 8.243)	7.338 (6.382, 8.061)	0.689	8.103 (7.796, 8.599)	7.836 (7.220, 8.385)	0.503
Post-AAD Mean DF (Hz)	6.986 (6.011, 7.677)	6.584 (5.801, 7.015)	0.014	6.732 (5.013, 7.534)	5.963 (5.430, 6.815)	0.039	7.225 (6.411, 7.781)	6.818 (5.859, 7.168)	0.002
Post-AAD COV-DF (%)	141.000 (104.000, 141.000)	141.000 (110.000, 141.000)	0.656	141.000 (116.500, 141.000)	141.000 (131.500, 141.000)	0.532	141.000 (96.600, 141.000)	140.000 (91.900, 141.000)	0.371

*DF, Dominant Frequency; COV-DF, Coefficient of Variation-Dominant Frequency.*

*Patients who did not sustain proper normal sinus rhythm and an AF status were excluded from the analysis.*

*Median (IQ1, IQ3) was displayed in the Table.*

*^†^n = The number of patients × AAD × Dose.*

### Termination or Defragmentation of Atrial Fibrillation Depending on the Dominant Frequency and Coefficient of Variation-Dominant Frequency

**FIGURE 4:**
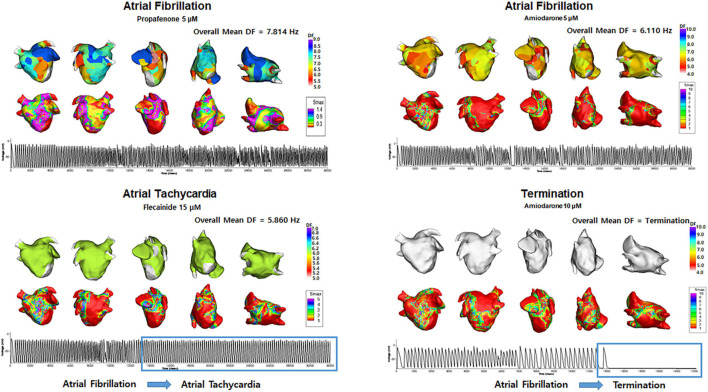
Changes in the dominant frequency (DF) during Termination and Atrial Tachycardia. The 3D DF map indicated that the mean DF was lower during AT episodes, and AF termination episodes. Electrograms demonstrated AT and AF termination episodes.

**FIGURE 5:**
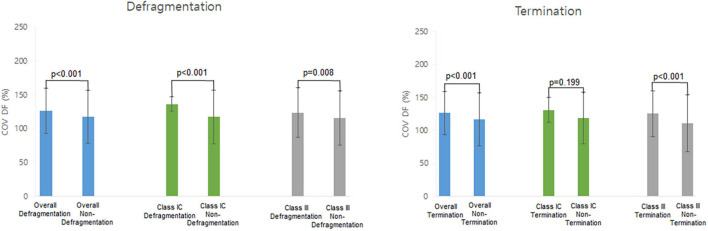
Heterogeneity of dominant frequency (DF). Heterogeneity of DF was observed in overall defragmentation as well as termination group.

Table 4 shows the electrophysiological characteristics of the termination and defragmentation episodes of AF after the AAD administration. In the episodes of AF defragmentation within 32 s after the AAD administration, the mean DF was significantly lower (*p* < 0.001, Table 4 and Figure 4), and the COV-DF was significantly higher (*p* < 0.001, Figure 5) than that in those with sustained AF. In the AF termination episodes, the mean DF was consistently lower (*p* < 0.001, Table 4) and COV-DF higher (*p* < 0.001, Figure 5). The tendency of a low DF and unstable (higher) COV-DF in the AF defragmentation episodes was consistent regardless of the class IC or class III AAD (Table 4).

**TABLE 4:** Electrophysiological characteristics terminated atrial fibrillation (AF) after antiarrhythmic drugs (AADs).

**Defragmentation**	**Overall AADs**			**Class IC**			**Class III**		
			
	**Defragmented (** ^†^ ***n* = 290)**	**Not defragmented (** ^†^ ***n* = 2210)**	***P*-value**	**Defragmented (** ^†^ ***n* = 60)**	**Not defragmented (** ^†^ ***n* = 940)**	***P*-value**	**Defragmented (** ^†^ ***n* = 230)**	**Not defragmented (** ^†^ ***n* = 1270)**	***P*-value**
Mean Smax	1.254 (1.022, 1.526)	1.263 (0.923, 1.675)	0.777	1.275 (0.920, 1.509)	1.271 (0.901, 1.549)	0.894	1.238 (1.036, 1.507)	1.255 (0.941, 1.856)	0.729
Mean DF (Hz)	5.476 (1.299, 6.706)	6.913 (6.233, 7.466)	<0.001	5.770 (5.201, 6.563)	7.118 (6.527, 7.860)	0.029	5.262 (1.299, 6.640)	6.710 (5.992, 7.227)	<0.001
COV-DF (%)	141.000 (139.000, 141.000) ^‡^126.166 ± 33.607	141.000 (108.500, 141.000) ^‡^117.571 ± 39.203	<0.001	141.000 (140.750, 141.000) ^‡^136.285 ± 10.847	141.000 (109.750, 141.000) ^‡^117.432 ± 39.783	<0.001	141.000 (138.250, 141.000) ^‡^123.734 ± 36.821	141.000 (98.625, 141.000) ^‡^115.311 ± 40.276	0.008

**Termination**	**Overall AADs**			**Class IC**			**Class III**		
			
	**Terminated (** ^†^ ***n* = 230)**	**Not Terminated (** ^†^ ***n* = 2270)**	***P*-value**	**Terminated (** ^†^ ***n* = 30)**	**Not Terminated (** ^†^ ***n* = 970)**	***P*-value**	**Terminated (** ^†^ ***n* = 200)**	**Not Terminated (** ^†^ ***n* = 1300)**	***P*-value**

Mean Smax	1.265 (1.041, 1.437)	1.263 (0.901, 1.675)	0.704	0.812 (0.809, 1.059)	1.264 (0.900, 1.552)	0.281	1.294 (1.089, 1.481)	1.255 (0.941, 1.856)	0.885
Mean DF (Hz)	5.295 (1.299, 6.677)	6.889 (6.170, 7.465)	<0.001	5.041 (3.170, 6.896)	7.101 (6.469, 7.843)	0.385	5.476 (1.299, 6.677)	6.170 (5.507, 6.684)	0.084
COV-DF (%)	141.000 (139.500, 141.000) ^‡^126.312 ± 33.185	141.000 (103.000, 141.000) ^‡^116.545 ± 40.083	<0.001	141.000 (136.000, 141.000) ^‡^131.124 ± 19.179	141.000 (113.000, 141.000) ^‡^118.718 ± 38.890	0.199	141.000 (141.000, 141.000)	138.000 (85.750, 141.000)	<0.001

*Smax, The Maximal slope of the restitution curves; DF, Dominant Frequency; COV-DF, Coefficient of Variation-Dominant Frequency.*

*Defragmentation: Termination + Atrial Tachycardia.*

*Patients who did not sustain proper normal sinus rhythm and an AF status were excluded from the analysis.*

*Median (IQ1, IQ3) was displayed in the Table.*

*^†^n = The number of patients × AAD × Dose.*

*^‡^Mean ± SD.*

## Discussion

### Main Findings

We evaluated the spatial changes in the AF wave-dynamics reflected by the mean DF and COV-DF after using AADs in a realistic computational model that reflected 25 AF patients’ LA geometry, histology, and electrophysiology. The AAD classes and doses apparently affected the AF wave-dynamics, and those effects differed between the PV and extra-PV regions depending on the Smax. The AADs easily caused defragmentation or termination at a reduced mean DF and spatially unstable DF (high COV-DF). Realistic AF computational modeling was a feasible approach to study the regional effect of AADs or electrophysiological changes.

### Anti-atrial Fibrillation Effects of Antiarrhythmic Drugs on the Pulmonary Vein or Extra-Pulmonary Vein Regions

The mechanism of the AADs involves the blocking of specific *trans*-membrane ion channels to inhibit the initiation or maintenance mechanisms of fibrillation. Class IC drugs function by blocking the rapid inward sodium current that slows the rate of the increasing action potential, and class III AADs block the outward potassium current, lengthening the repolarization and refractoriness (Kowey, 1998). However, it is not known how AADs act on different regions of the atrium and how the wave-dynamics react according to the anatomical structure. There have been many studies on the role of the PVs in the mechanism of AF (Khan, 2004). The PVs have a venous atrium origin that differs from that of other parts of the atrium in terms of the embryological development (Sherif, 2013) and are influenced highly by AF-associated genes such as *PITX2* (Wang et al., 2010). For this reason, the electrical isolation of the PV antrum is the most important target for AF catheter ablation (Chen et al., 1999). PV isolation blocks not only the triggers from the PVs, but also the cardiac autonomic nerves located in the PV antrum and reduces the atrial critical mass. In this study, AADs reduced the mean DF and its spatial instability (COV-DF) more significantly in the PV area than non-PV area. That suggested that the anti-AF effect of AADs mainly is responsible for the lower and spatially unstable DFs in the PV area than in the extra-PV areas. Investigation of the select effects of AADs on the LA regions can have a significant impact on the treatment of AF.

### Atrial Fibrillation Mechanisms of Multiple Wavelet or Focal Sources

The focal source hypothesis and multiple wavelet hypothesis have been considered as mechanisms of AF initiation and maintenance (Saad et al., 2009; Narayan and Jalife, 2014). The focal source hypothesis indicates that a special form of a reentry pattern of activation produced by rotors drives the AF mechanism. The multiple wavelet hypothesis explains the AF mechanism as spontaneous wave-breaks that constantly generate randomly wandering daughter wavelets. These wave-breaks collide, are disrupted, coalesce, or give rise to new wavelets in a self-sustaining turbulent process (Chen et al., 2000). High DF areas were used to locate the source of AF drivers or rotors (Hwang et al., 2016), and high Smax areas represent the vulnerable condition of AF wave-breaks in the AF maintenance mechanism (Kalifa et al., 2006). Therefore, the DF is a representative parameter for the focal source hypothesis, and the Smax advocates the multiple wavelet theory. Wu et al. (2002) reported that the focal source and multiple wavelets interact and maintain fibrillation according to the tissue conditions such as the conduction velocity. The present study demonstrated for the first time that the Smax has a direct effect on the DF wave-dynamics in AF and anti-AF mechanisms. Although the AADs did not decrease the Smax, the focal source mechanism represented by the DF was predominant in maintaining AF in atrial tissue with a low Smax. At a low Smax, the mean DF was high in both the PV and extra-PV regions, whereas the mean DF was low when the Smax was high. Therefore, the DF and Smax exhibited an inverse relationship.

### Sufficient Conditions for Atrial Fibrillation Defragmentation or Termination

Many studies (Pandit et al., 2005; Jarman et al., 2012; Sánchez et al., 2012) have been conducted over the years to understand spiral wave meander and AF termination in various ways. After the AADs, the continuous wave-breaks and reentrant behaviors could not be sustained, resulting in termination or defragmentation. Though a spiral meandering and reentry termination are challenging to study quantitatively (Pandit et al., 2005), we analyzed the DF and Smax changes during the AF defragmentation using realistic computational modeling of AF. This is because the computational modeling enabled spatiotemporally high-resolution mapping while repeatedly being performed (Li et al., 2016; Hwang et al., 2021). In this study, the changes in the DF wave-dynamics had a close relationship with the AF defragmentation. The defragmented AF episodes after the virtual AAD intervention exhibited a reduced mean DF and high COV-DF (spatial instability of DF) regardless of the type of AAD. These changes in the DF were consistently observed in the AF termination episodes. The change in the Smax did not have a direct effect on the AF termination, which was presumably because the AADs did not significantly change the Smax.

### Limitations

Right atrium (RA) was omitted from the study. The biatrial model is premature to be applied in personalized modeling because current image resolution cannot define the personalized interatrial connections. Heterogeneity due to nervous influence has been neglected. The fiber orientation layer was a monolayer. The LA wall thickness can be implemented to reflect a more clinically acceptable LA model. Bipolar voltage was not a feasible marker for fibrosis, and fiber orientation was not measured in a patient-specific manner. To incorporate a clinical electroanatomical map to the high-resolution computational modeling, we heavily extrapolated the limited number of bipolar electrograms. We measured DF at a fixed time window and it did not change over time. Regions especially PV specific ionic currents were not applied in this study due to lack of reference for ion currents effects of AADs on PV cells. No focal triggers were simulated in this study. The personalized LA model consisted of a monolayer. The LA wall thickness can be implemented to reflect a more clinically acceptable LA model. Multiple induction sites can reflect the complex mechanism of AF initiation (Prakosa et al., 2018). Although there are some differences in the rate-dependent action potential changes, restitution, and calcium dynamics among different human myocardial cell models (Nygren et al., 1998; Maleckar et al., 2009; Grandi et al., 2011; Koivumäki et al., 2011), the Courtemanche-Ramirez-Nattel model (Sossalla et al., 2010; Grandi et al., 2011) accurately represented the mathematical modeling of human atrial myocyte as indicated in our previous studies (Hwang et al., 2016, 2019, 2021; Lee et al., 2016; Lim et al., 2020a, b). The ion currents conductance values might not be an accurate representation of the effects of AADs in human atrial myocytes, however, the amount of uncertainty was minimal since large mammals were selected for references (Supplementary Table 2). Invasive mapping data were used for the analysis. Non-invasive late gadolinium enhancement of the cardiac magnetic resonance imaging data can be used for further analysis (Lopez-Perez et al., 2015).

## Conclusion

A DF reduction due to AADs is predominantly observed in the PV regions, and the AAD-induced low and heterogeneous DF condition during a high Smax condition was associated with AF termination or defragmentation. Personalized AF computational modeling provided evidence of how AADs exhibit anti-AF effects according to the atrial region or electrophysiological condition.

## Data Availability Statement

The original contributions presented in the study are included in the article/Supplementary Material, further inquiries can be directed to the corresponding author/s.

## Ethics Statement

The studies involving human participants were reviewed and approved by the Institutional Review Board of the Severance Cardiovascular Hospital. The patients/participants provided their written informed consent to participate in this study.

## Author Contributions

IH and ZJ contributed to the data, statistical analyses, and writing of the manuscript. J-WP contributed to the statistical analyses and data acquisition. O-SK contributed to the software programming and data acquisition. BL confirmed the data acquisition and references. JL provided support for the software programming. H-TY, T-HK, and BJ contributed to the clinical data acquisition and interpretation of clinical data. H-NP contributed to the study design, clinical data acquisition, data interpretation, and revision of manuscript. All authors contributed to the article and approved the submitted version.

## Conflict of Interest

The authors declare that the research was conducted in the absence of any commercial or financial relationships that could be construed as a potential conflict of interest.

## Publisher’s Note

All claims expressed in this article are solely those of the authors and do not necessarily represent those of their affiliated organizations, or those of the publisher, the editors and the reviewers. Any product that may be evaluated in this article, or claim that may be made by its manufacturer, is not guaranteed or endorsed by the publisher.

## Footnotes

1
http://www.rstudio.com/
