# Corrigendum: Spatial changes in the atrial fibrillation wave-dynamics after using antiarrhythmic drugs: A computational modeling study

**DOI:** 10.3389/fphys.2022.992000

**Published:** 2022-12-15

**Authors:** Inseok Hwang, Ze Jin, Je-Wook Park, Oh-Seok Kwon, Byounghyun Lim, Jisu Lee, Hee-Tae Yu, Tae-Hoon Kim, Boyoung Joung, Hui-Nam Pak

**Affiliations:** Yonsei University Health System, Seoul, South Korea

**Keywords:** atrial fibrillation, computational modeling, antiarrhythmic drug, dominant frequency, spatial changes

In the published article, there was an error in the **Author** list. Author “Ze Jin” was erroneously excluded. The corrected **Author** list appears below:

“Inseok Hwang, Ze Jin, Je-Wook Park, Oh-Seok Kwon, Byounghyun Lim, Jisu Lee, Hee-Tae Yu, Tae-Hoon Kim, Boyoung Joung, Hui-Nam Pak.”

In the published article, there was an error in Supplementary Table S1. The ion currents for baselines and AADs were described incorrectly. The correct Supplementary Table S1 appears in the Supplementary material.

In the published article, the **Author Contributions** were described incorrectly:

“IH contributed to the data, statistical analyses, and writing of the manuscript. J-WP contributed to the statistical analyses and data acquisition. O-SK contributed to the software programming and data acquisition. BL confirmed the data acquisition and references. JL provided the support for the software programming. ZJ contributed the clinical data acquisition. H-TY, T-HK, and BJ contributed to the clinical data acquisition and interpretation of clinical data. H-NP contributed to the study design, clinical data acquisition, data interpretation, and revision of manuscript. All authors contributed to the article and approved the submitted version.”

The corrected **Author Contributions** statement appears below:

“IH and ZJ contributed to the data, statistical analyses, and writing of the manuscript. J-WP contributed to the statistical analyses and data acquisition. O-SK contributed to the software programming and data acquisition. BL confirmed the data acquisition and references. JL provided support for the software programming. H-TY, T-HK, and BJ contributed to the clinical data acquisition and interpretation of clinical data. H-NP contributed to the study design, clinical data acquisition, data interpretation, and revision of manuscript. All authors contributed to the article and approved the submitted version.”

The authors apologize for these errors and state that this does not change the scientific conclusions of the article in any way. The original article has been updated.

