# Supplementary material for: Spatial Changes in the Atrial Fibrillation Wave-Dynamics After Using Antiarrhythmic Drugs: A Computational Modeling Study
Source: Front Physiol. 2021 Sep 24;12:733543. doi: 10.3389/fphys.2021.733543 (PMC8497701; doi:10.3389/fphys.2021.733543)
Supplement: Supplementary file 1 [file Data_Sheet_1.docx]

***Supplementary Material***

**Supplementary material reference**

Chen, K.H., Xu, X.H., Sun, H.Y., Du, X.L., Liu, H., Yang, L., Xiao, G.S., Wang, Y., Jin, M.W., and Li, G.R. (2016). Distinctive property and pharmacology of voltage-gated sodium current in rat atrial vs ventricular myocytes. *Heart Rhythm* 13**,** 762-770.

Delgado, C., Tamargo, J., Henzel, D., and Lorente, P. (1993). Effects of propafenone on calcium current in guinea-pig ventricular myocytes. *Br J Pharmacol* 108**,** 721-727.

Ducroq, J., Printemps, R., Guilbot, S., Gardette, J., Salvetat, C., and Le Grand, M. (2007). Action potential experiments complete hERG assay and QT-interval measurements in cardiac preclinical studies. *J Pharmacol Toxicol Methods* 56**,** 159-170.

Edrich, T., Wang, S.Y., and Wang, G.K. (2005). State-dependent block of human cardiac hNav1.5 sodium channels by propafenone. *J Membr Biol* 207**,** 35-43.

Gautier, P., Guillemare, E., Marion, A., Bertrand, J.P., Tourneur, Y., and Nisato, D. (2003). Electrophysiologic characterization of dronedarone in guinea pig ventricular cells. *J Cardiovasc Pharmacol* 41**,** 191-202.

Geng, L., Kong, C.W., Wong, A.O.T., Shum, A.M., Chow, M.Z.Y., Che, H., Zhang, C., Yau, K.L., Chan, C.W., Keung, W., and Li, R.A. (2018). Probing flecainide block of I(Na) using human pluripotent stem cell-derived ventricular cardiomyocytes adapted to automated patch-clamping and 2D monolayers. *Toxicol Lett* 294**,** 61-72.

Hilliard, F.A., Steele, D.S., Laver, D., Yang, Z., Le Marchand, S.J., Chopra, N., Piston, D.W., Huke, S., and Knollmann, B.C. (2010). Flecainide inhibits arrhythmogenic Ca2+ waves by open state block of ryanodine receptor Ca2+ release channels and reduction of Ca2+ spark mass. *J Mol Cell Cardiol* 48**,** 293-301.

Ji, Y., Takanari, H., Qile, M., Nalos, L., Houtman, M.J.C., Romunde, F.L., Heukers, R., Van Bergen En Henegouwen, P.M.P., Vos, M.A., and Van Der Heyden, M.a.G. (2017). Class III antiarrhythmic drugs amiodarone and dronedarone impair KIR 2.1 backward trafficking. *Journal of cellular and molecular medicine* 21**,** 2514-2523.

Lin, C., Ke, X., Cvetanovic, I., Ranade, V., and Somberg, J. (2007). The Effect of High Extracellular Potassium on IKr Inhibition by Anti-Arrhythmic Agents. *Cardiology* 108**,** 18-27.

Paul, A.A., Witchel, H.J., and Hancox, J.C. (2002). Inhibition of the current of heterologously expressed HERG potassium channels by flecainide and comparison with quinidine, propafenone and lignocaine. *Br J Pharmacol* 136**,** 717-729.

Seki, A., Hagiwara, N., and Kasanuki, H. (1999). Effects of propafenone on K currents in human atrial myocytes. *Br J Pharmacol* 126**,** 1153-1162.

Varela, M., Colman, M.A., Hancox, J.C., and Aslanidi, O.V. (2016). Atrial Heterogeneity Generates Re-entrant Substrate during Atrial Fibrillation and Anti-arrhythmic Drug Action: Mechanistic Insights from Canine Atrial Models. *PLOS Computational Biology* 12**,** e1005245.

Wang, Z., Fermini, B., and Nattel, S. (1993). Mechanism of flecainide's rate-dependent actions on action potential duration in canine atrial tissue. *J Pharmacol Exp Ther* 267**,** 575-581.

Wegener, F.T., Ehrlich, J.R., and Hohnloser, S.H. (2006). Dronedarone: an emerging agent with rhythm- and rate-controlling effects. *J Cardiovasc Electrophysiol* 17 Suppl 2**,** S17-20.

Yue, L., Feng, J.L., Wang, Z., and Nattel, S. (2000). Effects of ambasilide, quinidine, flecainide and verapamil on ultra-rapid delayed rectifier potassium currents in canine atrial myocytes. *Cardiovasc Res* 46**,** 151-161

**Supplementary Table 1. AAD Ion currents for Courtemanche-Ramirez-Nattel model**

|  |  | **^‡^CRN sinus rhythm** | | | | | | | | | |
| --- | --- | --- | --- | --- | --- | --- | --- | --- | --- | --- | --- |
|  |  | **Amiodarone** | | **Sotalol** | | **Dronedarone** | | **Flecainide** | | **Propafenone** | |
|  | **Baseline** | **5 uM (%)** | **10 uM (%)** | **60 uM (%)** | **10 mM (%)** | **3 uM (%)** | **10 uM (%)** | **5 uM (%)** | **15 uM (%)** | **5 uM (%)** | **10 uM (%)** |
| **gNa** | 100 | 111 | 105 | 95 | 111 | 111 | 100 | 93 | 56 | 86 | 62 |
| **gK1** | 100 | 81 | 72 | 95 | 95 | 90 | 72 | 95 | 95 | 95 | 95 |
| **gto** | 100 | 117 | 117 | 117 | 117 | 117 | 117 | 117 | 191 | 261 | 179 |
| **gKr** | 100 | 102 | 90 | 84 | 46 | 102 | 82 | 120 | 120 | 108 | 72 |
| **gCaL** | 100 | 250 | 225 | 150 | 150 | 200 | 120 | 150 | 215 | 235 | 190 |
| **gKur** | 100 | 200 | 200 | 100 | 100 | 100 | 100 | 120 | 100 | 162 | 96 |
| **gKs** | 100 | 144 | 128 | 144 | 128 | 128 | 96 | 160 | 160 | 160 | 160 |
| **INaCa (Max)** | 100 | 155 | 155 | 155 | 155 | 155 | 155 | 155 | 155 | 155 | 155 |
| **INaK (Max)** | 100 | 100 | 100 | 100 | 100 | 100 | 100 | 100 | 100 | 100 | 100 |
| **Iup (Max)** | 100 | 100 | 100 | 100 | 100 | 100 | 100 | 100 | 100 | 100 | 100 |
| **Krel** | 100 | 100 | 100 | 100 | 100 | 100 | 100 | 100 | 100 | 100 | 100 |
| **Caup (Max)** | 100 | 125 | 125 | 125 | 125 | 125 | 125 | 125 | 125 | 125 | 125 |
| **Ach** | 100 | 22 | 15 | 100 | 100 | 100 | 100 | 100 | 100 | 100 | 100 |
|  |  | **^‡^CRN AF** | | | | | | | | | |
| **gNa** | 90 | 90 | 85 | 77 | 90 | 90 | 81 | 75 | 45 | 70 | 50 |
| **gK1** | 210 | 180 | 160 | 210 | 210 | 200 | 160 | 210 | 210 | 210 | 210 |
| **gto** | 30 | 30 | 30 | 30 | 30 | 30 | 30 | 30 | 49 | 67 | 46 |
| **gKr** | 100 | 85 | 75 | 70 | 38 | 85 | 68 | 100 | 100 | 90 | 60 |
| **gCaL** | 30 | 50 | 45 | 30 | 30 | 40 | 24 | 30 | 43 | 47 | 38 |
| **gKur** | 50 | 100 | 100 | 50 | 50 | 50 | 50 | 60 | 50 | 81 | 48 |
| **gKs** | 100 | 90 | 80 | 90 | 80 | 80 | 60 | 100 | 100 | 100 | 100 |
| **INaCa (Max)** | 100 | 100 | 100 | 100 | 100 | 100 | 100 | 100 | 100 | 100 | 100 |
| **INaK (Max)** | 100 | 100 | 100 | 100 | 100 | 100 | 100 | 100 | 100 | 100 | 100 |
| **Iup (Max)** | 100 | 100 | 100 | 100 | 100 | 100 | 100 | 100 | 100 | 100 | 100 |
| **Krel** | 100 | 100 | 100 | 100 | 100 | 100 | 100 | 100 | 100 | 100 | 100 |
| **Caup (Max)** | 80 | 80 | 80 | 80 | 80 | 80 | 80 | 80 | 80 | 80 | 80 |
| **Ach** | 100 | 22 | 15 | 100 | 100 | 100 | 100 | 100 | 100 | 100 | 100 |

^‡^CRN: Courtemanche-Ramirez-Nattel

**Supplementary Table 2. References for atrial cell ion currents depending on AADs**

| **AADs** | **Reference** | **Animal/human model** | **Method** | **Ion current change** |
| --- | --- | --- | --- | --- |
| **Flecainide**  (5 μM, 15 μM) | Geng L. et al., 2018  Yue L. et al.(Edrich et al., 2005), 2000  Wang Z. et al., 1993  Hilliard FA. et al., 2010 | Human pluripotent stem cell-derived ventricular cardiomyocyte  Human right atrial appendage  Human pluripotent stem cell-derived ventricular cardiomyocyte  Canine, murine ventricular model | Whole-cell patch voltage clamp, microscope, and confocal laser-scanning unit | gNa,gKur,gNa,gto,gCaL |
| **Propafenone**  (5 μM, 10 μM) | Edrich T. et al., 2006  Paul AA. et al., 2002  A Seki. et al., 1999  Delgado C. et al., 1993 | Human Embryonic kidney cells  Human atrial myocytes  Guinea pig ventricular myocytes | Whole-cell patch voltage clamp | gNa, gto, gCaL,gKur, gKr, |
| **Amiodarone**  (5 μM, 10 μM) | Varela M. et al., 2016 | Canine atrial model | Microelectrode recording and patch-clamp | gK1, gKur, gNa, gKr, gCaL, gKs  Ach |
| **Sotalol**  (60 μM, 10 mM) | Ducroq J. et al., 2007  Lin C. et al., 2007 | Rabbit/Human embronic kidney cells  Xenopus oocytes | Bipolar Ag electrode recoding and patch clamp  Two-electrode voltage clamp | gNa, gKr, gKs |
| **Dronedarone**  (3 μM, 10 μM) | Chen KH. et al., 2016  Gautier P. et al., 2003  Ji Y. et al., 2013  Wegender. et al., 2006 | Rat  Guinea pig ventricular cardiomyocyte  Dog ventricular myocytes  Guinea pig myocytes | Whole-cell, perforated patch voltage-clamp | gCaL, gKs, gNa, gK1, gKr, gCaL |

**Supplementary Table 3. Baseline characteristics**

| **Characteristics** | **N=25** |
| --- | --- |
| Male, n (%) | 17 (68.0%) |
| Age, (Years) | 59.8±9.8 |
| <65, n (%) | 14 (56.0%) |
| 65–74, n (%) | 10 (40.0%) |
| ≥75, n (%) | 1 (4.0%) |
| Paroxysmal AF, n (%) | 8 (32.0%) |
| Follow-up Duration, (Months) | 14.2±15.3 |
| BMI, (Kg/m^2^) | 24.6±2.9 |
| CHA_2_DS_2_-VASc Score | 2.0±1.4 |
| Heart failure, n (%) | 3 (12.0%) |
| Hypertension, n (%) | 11 (44.0%) |
| Diabetes, n (%) | 4 (16.0%) |
| Stroke/TIA, n (%) | 5 (20.0%) |
| Vascular Disease, n (%) | 2 (8.0%) |
| Echocardiographic Parameters |  |
| LA Dimension, (mm) | 42.6±6.1 |
| LA Volume Index, (mL/m^2^) | 40.4±9.2 |
| LVEF (%) | 63.6±6.8 |
| E/Em | 10.6±3.8 |
| LA Voltage, (mV) | 1.9±0.8 |

BMI: Body Mass Index, TIA: Transient Ischemic Attack, LVEF: Left Ventricular Ejection Fraction, E: Early Diastolic Transmitral Flow Velocity, Em: Early Diastolic Mitral Annular Velocity.

**Supplementary Table 4. AF wave-dynamics depending on the Smax values**

|  | **Overall** | | **p-value** |  | **PV** | | **p-value** |  | **Extra-PV** | | **p-value** |
| --- | --- | --- | --- | --- | --- | --- | --- | --- | --- | --- | --- |
|  | **Smax < 1.4**  **(**^†^**n=13)** | **Smax ≥ 1.4**  **(**^†^**n=12)** |  |  | **Smax < 1.4**  **(**^†^**n=13)** | **Smax ≥ 1.4**  **(**^†^**n=12)** |  |  | **Smax < 1.4**  **(**^†^**n=13)** | **Smax ≥ 1.4**  **(**^†^**n=12)** |  |
| **Baseline**  **Mean DF, (Hz)** | 7.958  [7.138,8.485] | 7.708  [7.194,8.013] | 0.650 |  | 7.797  [6.246,8.243] | 7.338  [6.382,8.061] | 0.689 |  | 8.103  [7.796,8.599] | 7.836  [7.220,8.385] | 0.503 |
| **Post-AAD**  **Mean DF (Hz)** | 6.986  [6.011,7.677] | 6.584  [5.801,7.015] | 0.014 |  | 6.732  [5.013,7.534] | 5.963  [5.430,6.815] | 0.039 |  | 7.225  [6.411,7.781] | 6.818  [5.859,7.168] | 0.002 |
| **Flecainide 5 μM** | 8.203  [6.558-8.389] | 7.167  [6.348-7.419] |  |  | 8.115  [5.318-8.312] | 6.258  [6.013-6.955] |  |  | 8.419  [7.621-8.664] | 7.443  [6.366-7.594] |  |
| **Flecainide 15 μM** | 6.452  [5.436-6.920] | 6.707  [6.443-7.015] |  |  | 5.722  [4.697-6.711] | 6.815  [5.862-6.831] |  |  | 6.780  [5.970-7.237] | 6.816  [6.663-7.168] |  |
| **Propafenone 5 μM** | 7.730  [6.891-8.174] | 6.573  [6.127-7.019] |  |  | 7.585  [5.939-8.098] | 5.794  [5.723-5.866] |  |  | 7.842  [7.551-8.293] | 6.806  [6.249-7.363] |  |
| **Propafenone 10 μM** | 7.106  [6.332-7.166] | 6.828  [6.560-6.971] |  |  | 6.993  [4.899-7.093] | 6.820  [5.519-6.988] |  |  | 7.216  [7.175-7.327] | 6.943  [6.924-6.963] |  |
| **Amiodarone 5 μM** | 7.005  [6.251-7.213] | 6.060  [5.689-6.085] | 0.022 |  | 6.803  [6.040-7.032] | 5.281  [5.042-5.677] |  |  | 7.170  [6.390-7.385] | 6.124  [5.798-6.255] | <0.001 |
| **Amiodarone 10 μM** | 5.432  [3.316-5.584] | 5.705  [5.548-5.821] |  |  | 5.425  [3.312-5.469] | 5.292  [4.847-5.730] |  |  | 5.435  [3.317-5.625] | 5.877  [5.611-6.073] |  |
| **Sotalol 60 μM** | 7.551  [7.190-7.961] | 7.071  [6.294-7.200] |  |  | 7.345  [6.930-7.835] | 6.590  [5.732-6.971] |  |  | 7.774  [7.666-8.019] | 7.242  [6.287-7.455] |  |
| **Sotalol 10 mM** | 7.193  [5.262-7.673] | 6.880  [6.626-6.920] |  |  | 7.269  [4.119-7.433] | 6.082  [5.697-6.551] |  |  | 7.151  [5.239-7.787] | 7.011  [6.799-7.135] |  |
| **Dronedarone 3 μM** | 7.080  [6.824-7.462] | 5.890  [1.298-6.490] | 0.001 |  | 6.969  [6.530-7.220] | 5.606  [1.296-6.080] | 0.013 |  | 7.485  [6.964-7.586] | 6.122  [1.299-6.643] | <0.001 |
| **Dronedarone 10 μM** | 5.707  [3.863-6.288] | 5.967  [5.627-6.592] |  |  | 3.712  [2.220-5.794] | 5.361  [4.766-6.339] |  |  | 6.129  [4.399-6.378] | 6.146  [5.862-6.806] |  |
| **Post-AAD**  **COV-DF, (%)** | 141.000  [104.000,141.000] | 141.000  [110.000,141.000] | 0.656 |  | 141.000  [116.500,141.000] | 141.000  [131.500,141.000] | 0.532 |  | 141.000  [96.600,141.000] | 140.000  [91.900,141.000] | 0.371 |
|  |  |  |  |  |  |  |  |  |  |  |  |

DF: Dominant Frequency, COV-DF: Coefficient of Variation.

Note: Patients who did not sustain proper normal sinus rhythm and an AF status were excluded from the analysis.

Median [IQ1, IQ3]

†n= The number of patients *AAD*Dose

**Supplementary Figure legends**

**Supplementary Figure 1. Pacing and recording location of electrogram**

The representative electrograms were taken from the pacing site, which corresponded with the earliest activation site on the clinical local activation time (LAT) map during right atrial pacing (Bachmann's bundle activation site).

**Supplementary Figure 2. Overall 3D DF maps and high DF areas**

3D DF maps indicated overall DF, and high DF areas from baseline to AADs.

**Supplementary Figure 3. Overall 3D Smax maps and high Smax areas**

3D Smax maps demonstrated overall Smax, and high Smax areas from baseline to AADs
