# Supplementary figures and images for: Spatial Changes in the Atrial Fibrillation Wave-Dynamics After Using Antiarrhythmic Drugs: A Computational Modeling Study
Source: Front Physiol. 2021 Sep 24;12:733543. doi: 10.3389/fphys.2021.733543 (PMC8497701; doi:10.3389/fphys.2021.733543)

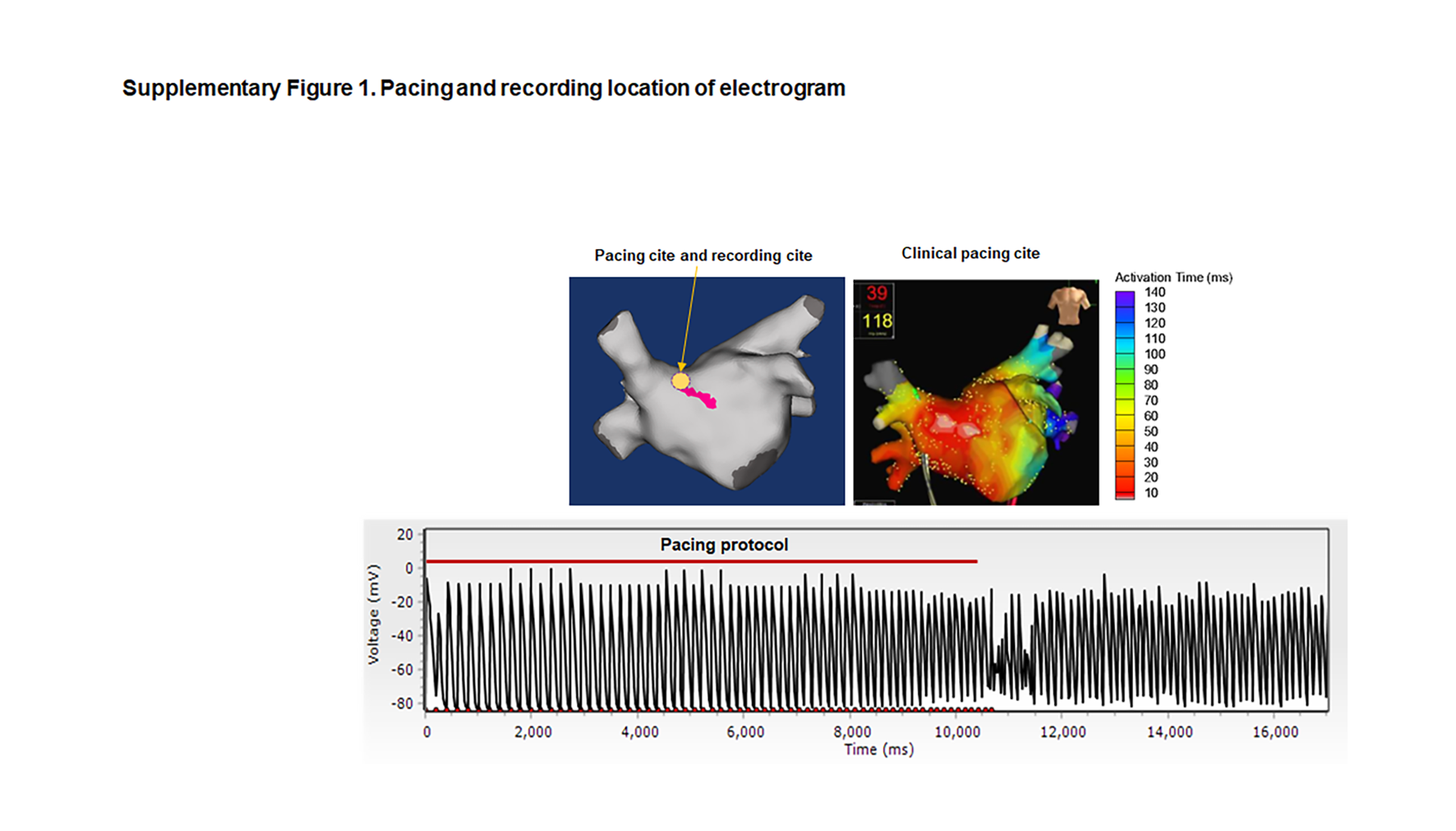

Supplement: Supplementary file 2 [file Image_1.TIF]

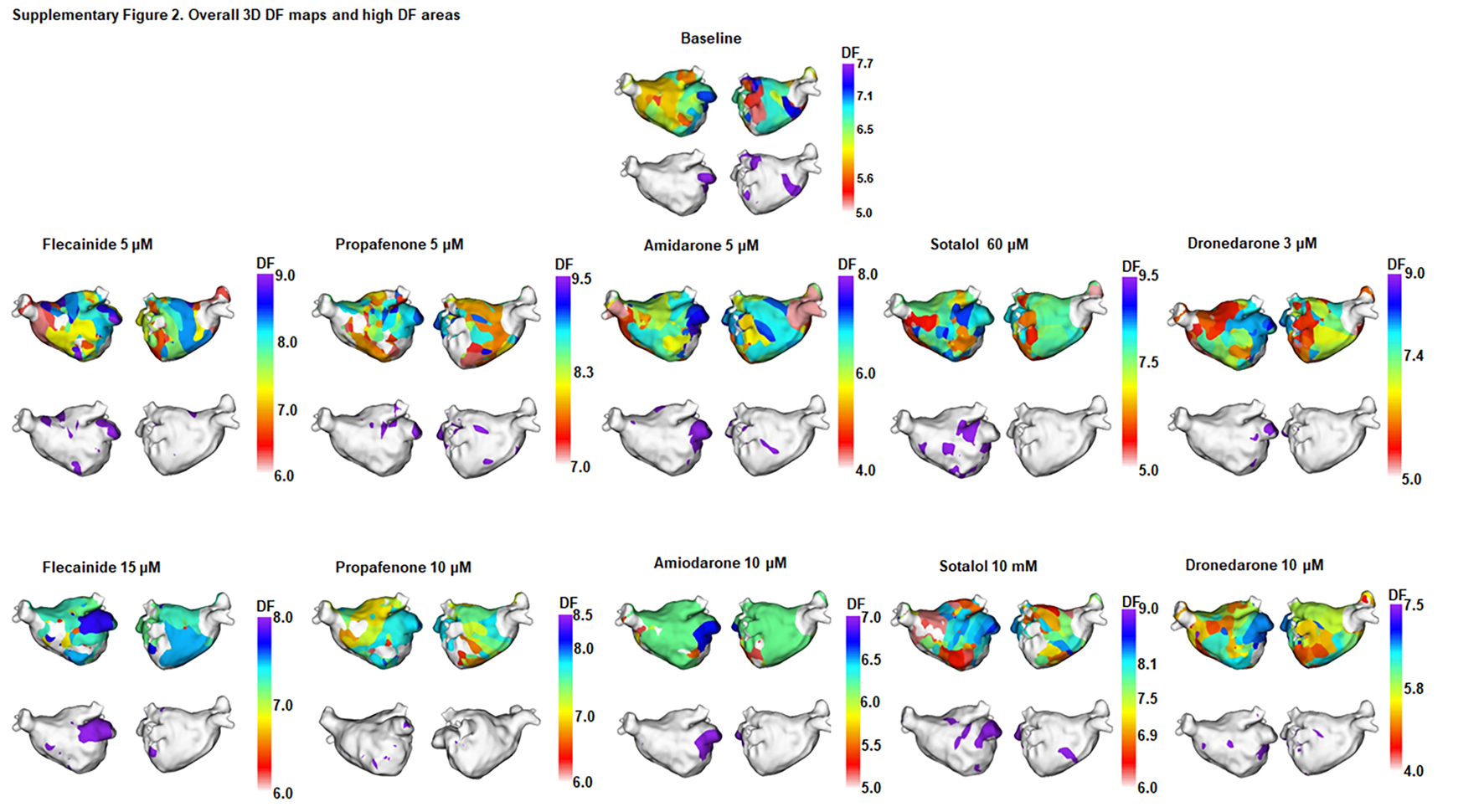

Supplement: Supplementary file 3 [file Image_2.TIF]

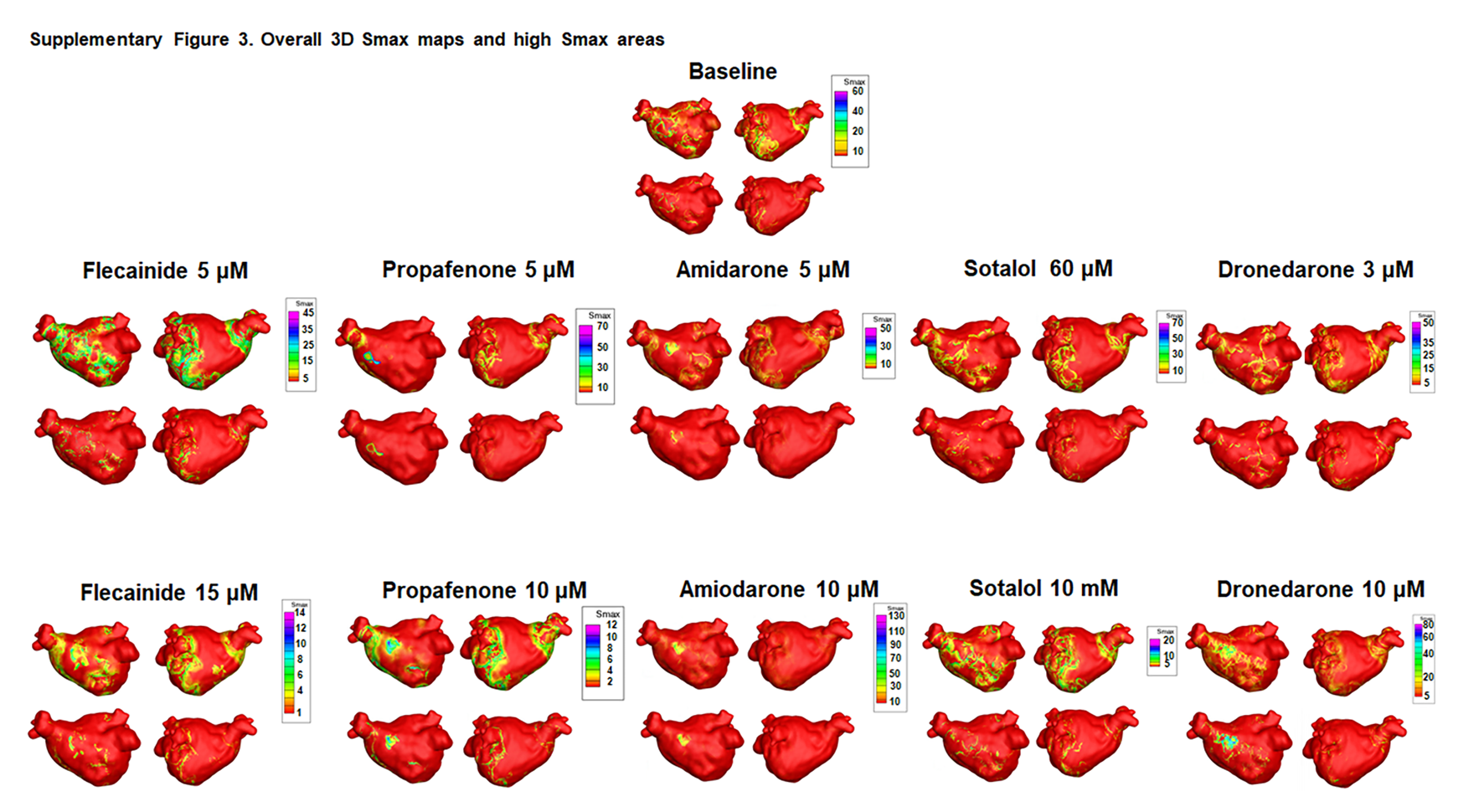

Supplement: Supplementary file 4 [file Image_3.TIF]
